# An “Ethical Moment” in Data Sharing

**DOI:** 10.1177/0162243916648220

**Published:** 2016-05-13

**Authors:** Catherine Heeney

**Affiliations:** 1 Science, Technology, and Innovation Studies, School of Social and Political Science, Edinburgh University, Edinburgh, UK

**Keywords:** ethics, methodologies, engagement, intervention

## Abstract

This study draws on interviews with forty-nine members of a biomedical research community in the UK that is involved in negotiating data sharing and access. During an interview, an interviewee used the words “ethical moment” to describe a confrontation between collaborators in relation to data sharing. In this article, I use this as a lens for thinking about relations between “the conceptual and the empirical” in a way that allows both analyst and actor to challenge the status quo and consider other ethical possibilities. Drawing on actor network theory (ANT), I approach “the empirical” using the concepts of controversy and ontological uncertainty as methodological tools to tackle the problem of ethics. I suggest that these concepts also provide a bridge for understanding the ontological structure of the virtual and the actual, as described in Deleuze’s *Difference and Repetition*. While other science and technology studies scholars have sought to draw on Deleuze, this article addresses the integration of ethics and empirical research. It arises as a critical reaction to existing treatments of this problem as found in empirical ethics, especially in the sociology of bioethics, and indirectly in ANT texts.

## Introduction

Biobanks and repositories that enable data sharing for the purposes of research have attracted social, ethical, and legal scholarship on governance, privacy, and informed consent (Laurie 2002, 2011; Tutton and Corrigan 2004; Boulton and Parker 2007; Haddow et al. 2008; Gottweis and Peterson 2008; Lunshof et al. 2008; Mascalzoni, Hicks, and Pramstaller 2009; Heeney et al. 2010). Sharing biomedical data presents actors with a shifting set of relationships to data, collaborators, and data subjects (Hilgartner and Brandt-Rauf 1994; Hackett 2005). In this article, I consider the task of bringing together the empirical and the ethical in relation to questions of data access and use. I argue that the empirical and the ethical do influence each other, and my aim is to make a case for the equality of analysts and actors in this process. I draw on forty-nine interviews with actors involved in using or setting up genetic databases in England and Wales between 2006 and 2009. I want to experiment with conceptual–empirical relationships (Jensen 2014) using the actors’ category of the “ethical moment,” which captures an event in which what is ethical comes into question. The ethical moment at the same time embodies an ontological controversy that allows both analyst and actor to consider ethical possibilities beyond practices and abstract moral frameworks.

Actor network theory (ANT) has engaged with ethics, in the form of care, for example, and appears broadly to take the stance that what is ethical is what is done in practice (see Mol 2008; Singleton 1996). What follows is a response to this stance. I consider the position of two fields that constitute explicit existing approaches to the problem of bringing ethics and the empirical together. These distinct approaches fall under the headings of sociology of bioethics, which has clear overlaps with ANT (Hedgecoe 2004; Pickersgill 2012; Wainwright et al. 2006) and empirical bioethics (Ives 2008; Parker 2007; Leget, Borry, and de Vries 2009; Dunn et al. 2012). Empirical bioethics sets out to use the empirical to enrich the analysts’ theoretical work as drawn from the field of moral philosophy; sociological work on ethics tends to focus on (and to varying degrees advocate) those carrying out practices (the actors), and not analysts, as the proper site of ethical judgment (Hedgecoe 2004; Wainwright et al. 2006; Cribb et al. 2008). My account differs from empirical ethics in that I am not directly trying to improve the application of existing moral principles or frameworks. However, I also seek to address a problem, which I see in sociological and science and technology studies (STS) accounts of ethics, where ethics is somehow found in actors’ discourse and practices without an accompanying account of the ethical on the part of the analyst, which might be examined and critiqued. Moreover, I see the claim that ethics *only* exists where it is being enacted or “deployed” as a barrier to the analyst having any ethical input. It may be that this is just a difference of opinion about how much to foreground the empirical, but as I will argue throughout the article this has important implications for when we as analysts try to discuss ethics.

Despite the differences between the approach I am advocating and ANT, ANT has provided invaluable tools for the analyst working with the empirical. One of these has been the highlighting of ontological ambiguity, as this suggests that “the real” is not fully present, which, I would argue, suggests it is not and cannot be fully captured in our observations of the empirical (Latour 2005; Law and Lien 2013; Law and Singleton 2005). This ontological uncertainty is explored through notions of contingency as revealed by “controversy” (Latour 2005) and in “studies of practical ontologies” (Gad and Jensen 2014). Therefore, while there are useful compatibilities between my approach and that of ANT, there is an important difference in that ANT (or ANT and its sympathizers) insists on “foregrounding practicalities materialities, events” (Mol 2002, 12). As a direct result, a given disease, for example, “becomes a part of what is done in practice” (Mol 2002, 12). However, I propose an account that does not privilege the empirical. Here I introduce the concept of “the virtual,” which suggests an alternative ontological structure to that adhered to by ANT. The virtual is neither totally abstract nor fully deployed or “actual,” and it can and does exert an influence, which is entirely real (Deleuze [1968] 1994). One example used to capture the idea of something being both virtual and actual is in relation to a gene, which “involves commanding several characteristics at once, and acting only in relation to other genes; the whole constitutes a virtuality, a potentiality” (Deleuze [1968] 1994, 234). The ethics I advance shares these qualities of virtuality and potentiality.

On the one hand, my use of a Deleuzian ontology echoes the rejection by ANT of an idealist position exemplified by Plato, for example, in which the empirical provides us with mere imperfect representations of pure essences or ideas. However, the virtual also works against the view that “the concrete is attained when the inadequacy of an abstraction is compensated for by the inadequacy of its opposite” (Deleuze [1968] 1994, 234). As ANT proponents would agree, a concept such as that of the gene, as with any other concept that is encountered empirically, is affected by an openness or indeterminacy. This is because it is linked with how the concept has been applied or could be applied in the past and future. However, the problem for ANT is that there is no possibility of gathering all these instances or applications together or making them present and thereby empirical (Patton 2000). Rather, some of them necessarily remain “elsewhere,” as Gad and Jensen (2014) have suggested. What follows is an exploration of a series of ethical moments, where interviewees describe conflicts regarding how to proceed ethically. That ethical moments arise because the relationships to elsewhere are not annulled (Delanda 2002, 75) is due to the influence of the virtual, which, I argue, provides the ontological grounds for the analyst to do ethics.

## Doing Ethics *on* the Empirical

Some proponents of the comparatively new field of empirical ethics suggest that knowing something of the circumstances in which a moral framework will apply is a valid and useful way of working toward its ethical application (Ives 2008; Ives and Draper 2009; Leget, Borry, and de Vries 2009). This can be done through understanding more about the practices and the circumstances in which ethical problems arise and may, perhaps optimistically, frame empirical work as a way to “lay bare the experiences, motives and intentions of those involved” (Leget, Borry, and de Vries 2009, 234). The “relevance of actual behavior” is simply “inescapable” (Sen 2009, 67). To organize “encounters with experience” (Ives 2008), therefore, appears to be a relevant preoccupation for the analyst interested in doing ethics. However, combining ethics with the empirical has long been recognized as an enterprise beset with conundrums, such as how and how far to distinguish between facts and values, and the barriers to deriving a normative imperative from a description of a state of affairs, or an “ought” from an “is” (Haimes and Williams 2007; Garrard and Wilkinson 2005; Ives and Draper 2009). The enormous difficulty of aligning a perspective that aims for universal solutions to ethical problems with one that aims at describing the particularities of a set of practices and circumstances has been well noted (Haimes and Williams 2007; Dunn et al. 2012). Unsurprisingly, the search for new combinations of the ethical and the empirical to address these problems continues (Dunn et al. 2012).

Empirical ethics enters a field equipped with a particular moral framework with the objective, broadly speaking, of comparing the empirical against this framework. However, the very notion of having a preestablished idea of what is ethical has drawn criticism from both the sociology of bioethics and the STS communities who see this as standing outside armed only with abstract principles, claiming an objectivity that nobody can have (Mol 1999; Singleton 1996; Wainwright et al. 2006). However, for analysts interested in engaging creatively with the ethical and the empirical, empirical ethics offers some helpful insights. I am persuaded that my job as an analyst is not to solve the actors’ controversies for them (Latour 2005). Nevertheless, a claim to recognize an ethical controversy, on the part of myself as an analyst, does I think require some sort of worked out system for describing how I process the ethical. Haimes (2002) has rightly pointed out that the relationships a person has with others will determine the extent to which a moral concept like autonomy coincides with her experience. Yet such a stance on the empirical enactment of autonomy rests upon an assumption that the analyst can distinguish between autonomy as described or observed and autonomy as it could or should be. At the empirical level, it seems fair to say that the analyst himself or herself confronts the ethical as a result of “situations in which, and by the extent to which, the moral is seen to be problematic, contested, in need of deliberation, analysis, or critique” (Parker 2007, 2255). It is this combination of the moral, the ethical, and the empirical that allows a distinction to be made between principles and frameworks (the moral) and a simultaneous recognition of and distancing from these frameworks in response to conflict (the ethical) Parker (2007). The ethical could be seen then as a potentiality (or a virtuality) not captured by an instance of the moral and the empirical.

## Ethicists and Actors

A more sociological distinction has also been made between what “principles and other normative frameworks suggest ought to be done” and what actors themselves feel to be correct or “deemed socially acceptable, good or right” (Wainwright et al. 2006, 734). In this socially grounded account of ethics, it is actors who distinguish the normative or the moral from the ethical, being aware of principles but using their own judgment in relation to their practices. Pickersgill (2012, 582) argues for the influence of a “regime of normativity,” which, he claims, provides conditions for both the production and questioning of ethical discourse. However, practice remains in the foreground via the claim that it is through practice that attempts are made by actors to “reinsert care into research” (Pickersgill 2013, 38). Moreover, the choice to foreground practice does not in itself answer the question of what makes the analyst think they are seeing ethical practices. Barnes (2001) suggests something extrapractical is needed for explanations based on practices to make sense (see also Gad and Jensen 2014). In the case of ethics, an analyst finds or recognizes something in the practices or discourses of informants, which is ethical but not normative. Moreover, a practice-based account ostensibly enables an analyst to avoid any appeal to normative or moral frameworks by describing actors’ ethical or care activities as being embedded or coproduced. However, foregrounding empirically observable practices in order to avoid charges such as essentialism, delusions of objectivity, or idealism, itself has been critiqued as foundationalist and reductionist (Turner cited in Gad and Jensen 2014; Delanda 2006).

## Ethics in Practice

Beyond a simple foregrounding of practices, a rejection of the role of the professional bioethicist in the production of ethical practices also appears in some sociological and STS accounts (Hedgecoe 2004; Mol 2008). The task of *doing* ethics properly falls to those actors involved in decisions around “every day,” “local” practices (Cribb et al. 2008; Pickersgill 2012; Haimes 2002; Hedgecoe 2004). Actors appear as “the genuine applied philosophers, working through a moral dilemma, using values and beliefs about morality to reach a decision that they then have to put into practice” (Hedgecoe 2004, 137). Those involved in practice (the actors) are those who decide which of their values apply best in a given situation. Mol asserts that in relation to the care of people with diabetes, “the crucial moral act is not making value judgments, but engaging in practical activities” (2008, 75). Capturing these ethically infused statements and actions of the actors is what provides the analysts with a “socially embedded ethics” (Cribb et al. 2008, 351). This has been contrasted with “a position of abstract rationality” rooted in a philosophical tradition (Wainwright et al. 2006, 745). This ethics *in* practice perspective is often accompanied by a more or less explicit suggestion that analysts *ought* to avoid doing ethics. Singleton, for example, talks about a “guilt-inducing discourse of should, which seems to be based in a discourse of oppression and domination” (1996, 462). Although Singleton is addressing a specific case, the way her conclusions were framed suggests that the term “should” lends itself to oppression and domination. Some have argued that revealing contingencies and contradictions, as STS does, is itself political and this makes explicit ethical engagement on the part of the analyst unnecessary (Gad and Jensen 2014). While this claim may be true in some cases, claims that ethics are simply found in practice and reducible thereto removes the grounds for the analyst to suggest alternatives.

## Ontologies of ANT

Mol argues that “reality does not precede the mundane practices in which we interact with it” (1999, 75). ANT has stressed the analytical usefulness of the ontologically uncertain or problematic: something such as a disease can be in many places at the same time but changes in significant ways as it enters into configurations with different discourses, people, technologies, and practices (Mol and Law 1994; Mol 2002; Law and Singleton 2005). ANT has given us the “fire object” which encapsulates “sets of present dynamics generated in, and generative of, realities that are necessarily absent” (Law and Singleton 2005, 343). However, a protocol, which is intended as a universal guide to behavior, “rests on real time work” (Timmermans and Berg 1997, 275). What happens is determined in the local context by the institutional, the infrastructural, and the material. Therefore, the virtual has no role in this account of “local universalism” (Timmermans and Berg 1997). This renders rhetorical a question such as that raised by Mol regarding practices of “care”: “they deserve to be able to travel but how?” (2008, 87). How can we respond to something deserving to travel or indeed the call for STS to do more than debunk (Latour 2004), if the analyst can only engage with what is already “completely deployed in their relations with the world” (Harmon 2009, 18)?

## Controversy and Ethics

Latour states that “there is no rear-world behind to be used as a judge of this one” (2005, 118). Latour’s early rejection of an idealist position is evident in statements such as “There is no pre-established harmony” (1988, 164), from where he moves to the statement that “How something holds together is determined on the field of battle” (1988, 164). Despite ANT’s interest in controversy, the reluctance to engage with ethics is arguably bound up with the ontological position that there is nothing but that which is somehow deployed. There may be no preestablished harmony or at least not one that provides us with any useful epistemological tool *or* moral framework. However, it does not follow that *which is* is only that which is deployed or actual. The ontological controversies that interest me are not only about scientists’ claims about what *is*, they are also about what should be. The virtual creates conditions for actual experience, not as a preestablished harmony but as “a changing series of relations” (Williams 2013, 9), that disrupts and troubles the actual. Therefore, while Latour’s (2005) advice about being attentive to controversies is not intended to be applied thus, it is potentially productive for analysts who hope to engage with both ethics and the empirical. It is “a way in” for the analyst to consider not only questions such as “Why is this problematic?” but also “What would you do differently?” As Fraser points out, it is in “recognising the role that social scientists play in creating the worlds they seek to investigate and… wishing to change worlds that include more than social scientists and their objects alone––that the concept of the virtual is of value” (2010, 77).

## Ontology and Ethics

Terms like “regime of normativity” (see Pickersgill 2013, 582) show the difficulty of capturing the conditions for the ethical by reference to actual practices alone. A regime of normativity has “a part to play in the structuring of everyday work” (Pickersgill 2013, 582), but it is not fully expressed in or reducible to practice. Nor is it entirely findable in a particular material object like, for example, a written set of normative guidelines. Practices and objects gain significance due to their being “irremediably entangled with discourses and histories produced in other places––elsewhere” (Gad and Jensen 2014, 11). This elsewhere disrupts and challenges the actual but is not a transcendental fixed moral state to which the actual provides a poor comparison. “Morality … is the system of judgement. But ethics overthrows the system of judgement” (Deleuze 1988 in Bryant 2012, 33). Therefore, while the “moral” attempts to impose a fixed external standard, ethics is a creative and transformative process, the means of bringing about something new (Buchanan 2011). What is normative in Deleuzian ethics, therefore, is the ability to critique, transform, and create (Jun 2011). ANT gives us the fire object, but what about its inverse––the *absent* dynamics––that are generative of realities that are present? Such dynamics are an important part of an ethical moment.

## Interviewing on Data Sharing Practices and Ethics

As part of a project on the governance of biobanks, I carried out forty-nine semistructured research interviews between 2006 and 2009, with individuals involved in the management of “genetic databases” (or “biobanks”) in the UK. The biobanks in this project included a variety of organizational forms, from small tissue and sample collections, to repositories destined to serve the research community, to genomic data sets. People working in a range of capacities were interviewed. While the majority of interviewees were directly involved in research in a full- or part-time capacity, nine interviewees were primarily occupied with the facilitation of research for the wider scientific community. Twenty-one interviewees had a clinical role at some time as well as being active in research. The project team drew up an interview schedule. All interviewees were asked in the first instance both about their professional background and about how they had collected the samples and data with which they were working. They were then asked more detailed questions about governance, storage, and the sharing of data (see Appendix). A professional transcriber transcribed the interviews verbatim. A set of codes were devised by the author with the intent to draw out the interviewees’ statements about their ethical and moral reflections on the data sharing and collaborative aspects of their work. These data were coded using Nvivo 7. My aim here is to provide examples of controversy arising in the context of data sharing or changes in access to data. The focus will be on how interviewees talk about challenges and relate these to what they see as ethical requirements and normative frameworks.

Data sharing via biobanks and collaborations is necessarily a geographically dispersed activity, and, in some cases, only a part of what the interviewees were doing locally. In many cases, there were not yet regularities of behavior or routine (Rouse 2001). As we shall see below in what the actors say about practices, “what past and present practice *is* includes its possible futures, which have not yet been fully determined” (Rouse 2001, 197). Data sharing in the majority of the examples I give below posed a challenge to what people had been doing or prompted discussion about what they would like to do. I am interested in the discourses interviewees employ and create, and I use the interview data to consider what they think ought to be considered in relation to what is and is not ethical in terms of practice. Therefore, interviewees appear not only as “*practicing actors*” but also as “*thinkers*” who relate their desires for their own practices with the wider normative, scientific, and social implications of what they do (Gad and Jensen 2014). One of the questions of interest was how to understand what was ethical, when new forms of interaction and new relationships were being constructed within a new assemblage. I do not attempt to distinguish between the *real* ethical position and interests of the interviewees.

## Ethics and Governance: Controversies and Practices

As discussed, the normative, understood as a formal and external framework, has been contrasted with what actors see as being “socially acceptable, good or right” (Wainwright et al. 2006, 734). The following examples are cases where this distinction appears to be born out as interviewees contrast their own ethical vision with a rigid governance structure that does not allow responsiveness to what is *really* ethically important about practice. The following interviewee was a clinical neurologist as well as a research scientist. As part of a dialogue on obtaining consent for patients to be involved in research, the interviewee repeated the theme of frustration with the ethics committees’.^1^

Well, I had a conversation with that patient where it was quite clear to me they wanted me to look for the cause of their disease so I should carry on looking. Should I ask the ethics committee every time I want to screen a gene? (Interview 14, Clinician/Researcher)

The interviewee evoked a relationship between patient and doctor in order to argue for the ethics of research. This relationship is used by the interviewee to argue that what is at issue ethically is not what the ethics committee focuses on. The conflict between the ethics committee and the interviewee is an ontological one at the same time, as it is a controversy about the ethical meaning of testing new genes in connection with a disease. The interviewee claimed that tests of different mutations were really different instances of the *same* thing, namely, the search for the genetic mutations implicated in the disease affecting his patients. In this context, therefore, screenings for different genes are both ontologically and ethically the same. Therefore, for the interviewee, the ethics committee is wrong to see each screening for a new gene as ontologically and ethically separate. In this regard, the relationship with the patient, and the conversation with this patient, is presented by the interviewee as having more authentic ethical authority. A perceived inability to grasp ethically important ontological distinctions also prompted the clinician in the next excerpt to complain that Local Research Ethics Committees (LRECs) had impeded access to National Health Service (NHS) data, which had in turn impeded his research. LRECs are criticized for being remote––both in a geographical and in an ethical sense––to the patients and data subjects, who he evokes as being sympathetic to his aims.

Research ethics committees that haven’t got a clue about the ethics that people in their council estate where I’ve been this morning in [area], care about. (Interviewee 41, Clinician/Researcher)

On the face of it, this supports the STS and sociological understandings of ethics that the scientist sees what is ethically important due to being embedded in practice. These interviewees challenge the moral authority of the ethics committee by drawing on what patients or patients’ communities wanted. Another interviewee claimed, his research had been “held up by the unethical nature of ethical [*sic*] committees” (Interview 44, Clinician/Researcher), because there were local approaches to assessing research access to NHS patients. The challenge to the moral authority of LRECs is prompted by the limits placed upon the interviewees’ research opportunities but also by differing ontological and ethical stances. The interviewees themselves present a picture of their own embeddedness in practice as conferring authority on them to make ethical claims. However, the practices the interviewees refer to aren’t precisely *actual* practices, but rather *potential* practices that are dependent on taking a different position than that which ethics committees take. While these examples on one level support the dichotomy between an ethics in practice and abstract normative frameworks, they also challenge the notion that practitioners’ ethics are embedded in practice.

## Negotiating the Ethical

Biobanks are in many cases repositories, that is, institutions that store data in order to facilitate access to a broad range of potential users. For many of the interviewees, these were fairly new configurations of the material, the professional, and the organizational, and they were full of ontological controversies that were at the same time ethical controversies. One explanation given for this was that actors coming into a biobank were from different professional disciplines. In the case of one set of information technology (IT) professionals, a practice taken for granted by them became an ethical challenge, which confronted not only their understanding of what they were doing but also that of the scientists and the ethics committee. Creating “backups” entails ensuring that data sets are copied, so that damage to the original will not entail loss of the data. The ethics committee and scientists wanted to allow individual participants to withdraw from the study and for this promise of withdrawal to form part of the informed consent process. However, for the IT professionals,^2^ the practice of “backing up” was at odds with the promise of withdrawal, as the individual’s contribution to the data would still be included in the backup copies of the data set. Below is an example of how they responded to being asked how they had negotiated with other groups involved in creating the biobank.

We talked through the principle of physically how do you deal with withdrawal of consent in that back up environment …. But now we’re into okay but we agreed when a restore was done that people who had withdrawn consent their data would be destroyed, now we’re into a debate about what it is destruction. (Interview 40, IT Professional)

This conflict led the IT professionals to suggest that their role had been understood as purely technical and operational. As they put it, they were the “Cinderella section.” However, the withdrawal and backup problems made it clear to them that “the science community hasn’t got the basic understanding of IT and the implications of it” (Interview 40, IT Professional). This situation is difficult to attribute to an opposition between a rigid normative framework and what the scientist sees as “good.” An external normative framework, which could be contrasted with one relatively more internal to the scientists and their practices, did not yet exist. In important ways, the scientists were aligned with the ethics committee, who were feeling their way in this new assemblage, along with a variety of other actors (including epidemiologists and clinicians) and actants (such as the backup files). This interesting coming together was best revealed by attending to what the disagreement was about (Latour 2005). Moreover, attending to this disagreement opens the door for the analyst to consider what the ethical alternatives could be in this dilemma—just as the actors are doing. It is interesting that the IT professionals wanted to shed their Cinderella status, as they perceived it but it is also interesting to see how this discussion about withdrawal and backups impacted the discussion on ethics (see Laurie 2011). The story prompts the analyst to recognize that ethics cannot be reduced to a rigid normative framework *or* to practices. This challenging event or controversy does not highlight a dichotomy but rather raises many ethical possibilities—for actors and analysts, it “presupposes a swarm of differences” (Deleuze [1968] 1994, 61).

## Ethics and the Enrollment of Governance

Ethics or other forms of “non-science” can maintain an image of science in a way that may even “deprivilege” science (Wainwright et al. 2006, 773). ANT might alternatively see this as a drive to enroll ethical frameworks (Callon 1986). In the example below, an epidemiologist is comforted by the fact that the chairman of an LREC agreed that it is ethical to perform a meta-analysis on available data.^3^ This agreement arose due to the LREC chairman being willing to hold informal discussions with the interviewee in relation to a particular ethical issue that of “pooling” data from different existing clinical trials.

So we’ve asked the ethics chairman before whether they think there’s a need for ethics approval for that sort of project. And I’m not the only person doing this sort of work––it’s international and the general feeling is no because the meta-analysis, the pooling of these data is about answering the same question as the data we originally collected for in these individual studies, it’s just answering it with more precision because you’ve got more data. (Interview 18, Epidemiologist)

The LREC chairman was enrolled alongside the “general feeling” among a scientific research community to constitute the practices as ethical. However, the interviewee also described an alternative scenario, where DNA is studied irrespective of its connection to a particular disease as requiring more formal ethics committee scrutiny.

But if they’re looking at different questions to the ones that we were asking then I might be concerned that they actually need ethical [*sic*] committee approval because we’ve never, for example looking at the DNA in its own right for a different genetic mutation has got nothing to do with the condition we were looking at. (Interview 18, Epidemiologist)

This is arguably an instance of ethical boundary work (Wainwright et al. 2006; see also Gieryn 1983) or enrollment of actors based in ethics. There are two interesting points to make based on the position this interviewee takes. On the one hand, there is again a difficulty in separating a rigid external ethics from what the interviewee does in practice herself. On the other, while she is describing ethics as something being constructed by a set of actors, she resists the idea that another set of practices could find a similar route to being thought of as ethical. Rather, her claim suggests there is a notion of the ethical that individuals should not take for granted even in the face of a possible consensus about how to act. In the example above, the alternative scientific proposal she considers would need to face formal ethical scrutiny. This again suggests that there is something about the ethical that is more than just the point scoring instrumentalism of boundary work. So even enrollment of the ethics committee chairman and other scientists, as described in the case of pooling data, does not for the interviewee in itself make something ethical.

## Consent and Practice

The practice of gaining informed consent in order to embody the abstract principle of autonomy (Faden and Beauchamp 1986) was pervasive in activities around biobanks. However, as we have seen already in some of the examples above, data are often mediated by the relationships researchers have with patients and their families through their clinics. So one clinician, who worked on rare genetic mutations involved in heart disease, stated that consent allowed him and his team to operate “fairly freely.” He also noted that families have less interest in the “niceties of consent and privacy and data coordination” (Interview 12, Clinician/Researcher) than in progress in explaining their condition. This relationship to consent can be understood in the context of the clinician’s experience and familiarity with the families whose data he and his team were analyzing. In many of the new biobank scenarios, the clinician working with families will no longer be directly involved when other researchers use the data. Thus, the link between the patient and the clinician is an absent dynamic (see Law and Singleton 2005). Indeed, for the data subject, the loss of the link with the clinician means that informed consent also has the function of a decree absolute, effectively divorcing (and excluding) them from further input in the research process.

This function of consent could be seen where the data were to be held in a repository and the original collector, and, indeed, their connections with the data subjects were to be “effaced” (Lee and Stenner 1999). Yet absent dynamics in many cases do not fully submit to this process of effacement and instead appear as ethical considerations. The next interviewee, who has much experience working with large repositories of data from different nonhuman organisms, talks about the problems of privacy and anonymity. The challenges to keeping practices ethical, according to the justifications the interviewee is familiar with, arise due to the introduction of human data subjects. These issues remain defiantly present in the face of the positive messages about the benefits of open access for genomics research (Heeney et al. 2010).

And the experience in genomics again and again is that datasets are valuable when they’re aggregated together and … when people have the freedom to think about any sensible obviously ethical in this case, I mean any sensible use of that data. (Interviewee 10, Bioinformatician)

Due to the interviewee’s belief in the scientific value of open access, he describes having dismissed resistance from clinical colleagues as being instrumental, a mere matter of protecting one’s own research territory. However, the interviewee finds that the ethical issues raised on occasion by clinicians and other scientists (who are often the original collectors of the data) cannot be canceled out either with claims of scientific protectionism or by recourse to informed consent. The interviewee claimed that despite his best efforts to reduce them to self-interest on the part of scientific and clinical colleagues, the tensions between the benefits of open access and the ethical issues persisted in causing him an “annoying headache.”

## Consent and Uncanceled Dynamics

Attempts to employ informed consent as a tool to future-proof uses of the data against ethical challenges was a recurring theme. Yet ethical uncertainties remained, as in the example above. The following interviewee was a repository-based IT professional who admitted that using a broad consent to free the data for a wide variety of uses failed to capture fully some ethically important aspects of the data. The interviewee claimed that this was due to the fact that data had been generated for other purposes by other users in other settings. In other words, the data had come from elsewhere.

And have they been obtained in the same manner with the same donor consent, in the same spirit that we do that here and if so how could we guarantee it and how could we then you know go on to pass it on to and be responsible in a sense for its quality when it goes further down the line to researchers? (Interview 35, IT Professional)

In addition to the new repository style biobanks created from scratch, some repositories subsume data from existing studies and allow them to be used by the research community, broadly defined. This happened with several cohort studies, which in some cases housed data collected over the life course of a group of individuals. The integration of such studies within new repository assemblages involved confrontation between different sets of practices and discourses, as we have seen above. One interviewee described the evolution of the cohort study, which he had been heavily involved in maintaining, into a resource to be used in a repository-type setting. Here, he describes his discussions with his local ethics committees about this process.

Then we had to discuss the arrangements for the future and … that’s an area which is very important it seems to me is having got ethical approval for the original field study, it doesn’t necessarily mean you’ve got ethics approval for every possible use of the biological material. (Interview 15, Clinician/researcher)

Increasingly, data sharing involved the practice of making data open access via a repository. Yet, as with informed consent above, the interviewee felt that ethics committee approval did not capture the modes of access in a “large biobank,” wherein data would be openly available for a variety of research uses by a variety of users. The interviewee suggested that parts of the normative or governance framework were being challenged by these developments and competing drives rooted in different ontological visions of the data resource.

I think the real tension that is arising is between a model which is built around the … free and potent sharing of genetic sequence data and a model which is much more rooted in the clinical trials and long term follow up studies. (Interview 15, Clinician/Researcher)

This ethical tension related again to an ontological disagreement about what the data were. It was also intimately connected to the interviewee’s diminishing role as a gatekeeper, which he explicitly referred to elsewhere in the interview. The ontological status of the data is not settled, connected as it is to some of those involved in an “original field of study.”

An interviewee who described himself as someone who was brought in “to make sure the computers worked” (Interview 30, IT consultant) had a role in managing data storage and access for a variety of projects within one institution. The following is part of an anecdote employed as an example of the type of governance problems he had confronted in this role. The story related to a collaborating organization that wanted to use the data to study a disease but also to link back to particular individuals in order to invite them to participate in a targeted piece of research. The interviewee again felt that there were expectations about participation that were not matched by this particular use of the data.

Yes I think that was definitely a sort of ethical moment because they were saying, well you know you’re not going to get your funding if you don’t do this and we were saying yes, yes. So it’s quite a, and the lack of, the lack of any I mean obviously there’s no law on that. (Interview 30, IT Consultant)

This challenging event arose from a confrontation between different understandings of what was ethical in relation to the use of the data. Again, the interviewee’s position was influenced by an earlier situation or absent dynamic. The interviewee said that when participants in the study were recruited, they “weren’t signed up to be approached” in this way. The ethical moment evoked by this interviewee, in common with the tensions noted by the interviewees quoted above, is a conflict in which existing guidance does not adequately capture what is happening. Moreover, an ethical solution does not present itself to the interviewees due to their being embedded in practice. This suggests that the ethical is an idea or a goal, reducible neither to practice nor to a given moral framework. Rather, it is a creative response to the sorts of dilemmas with which the interviewees grapple.

## Discussion

With the concept of the ethical moment, I have challenged a dichotomous view of ethics as either practices or guidelines. The ethical moment was an actors’ category that captured the struggle to bring ideas of the ethical together with unfolding practices. The ethical moment is an event whose significance I have explored in terms of both ethics and ontology. I have argued that ethics partakes of the virtual and, therefore, is not something fixed or universal but a changing series of relations, which challenges both fixed ideals and actual practices (Williams 2013). A new data sharing arrangement may employ informed consent to settle differences or to avoid conflicts, yet controversy remains, tensions are not canceled (Delanda 2002). These tensions are visible, where actors engage in “deliberation, analysis, or critique” (Parker 2007). Following the advice of ANT, I have been attentive to controversies where actors challenged normative frameworks or voiced concern that something was missing from them. The controversies, which often involve moral language to defend or criticize a position, were an opportunity to consider the ethical issues raised by, and therefore of relevance to, the actors. These erupting tensions, which are the source of controversy and disagreement, happen despite the many concrete attempts to construct and fix practice as ethical. Equally, they were a “way in” for me, the analyst, enabling me to be aware of and engage with these issues. Therefore, my perspective, while not the same as those of the actors, cannot be dismissed as coming from a fixed abstract framework untroubled by empirical insights.

Attempts to circumvent or remove existing “gatekeepers” or “obligatory passage points” had been particularly contentious for many of the interviewees. Local disagreements about ontology and ethics continued despite the use of informed consent to cement new configurations of the ethical and the material. These disagreements are reminders of the gatekeepers and the relationships to elsewhere that they evoke (see Gad and Jensen 2014). I focused particularly on the approval of ethics committees and the obtaining of informed consent, as examples of moral or normative mechanisms. Informed consent is an issue that has been alive for many years in the research community, but it continually shifts with its role in new assemblages. Consent is seen as necessary to release the data to the repository because, as Star and Griesemer (1989) note, repositories remove the need for negotiating new uses for the resource with the original collectors (or research participants). The signed informed consent form is thus intended to be part of a “standardized package” (Fujimura 1992), which comes along with the data and fixes subsequent uses as ethical, thus canceling disagreements in advance. Yet the controversies the actors themselves talked about indicate an “overstretching” of informed consent (Corrigan 2004), which links to debates in bioethics around broad informed consent as an attempt to make future and unknown practices ethical (Hofman 2009; Sheehan 2011). In the examples above, the informed consent and ethics committees played a complex role, which cannot be reduced to an abstract moral framework external to practice.

Work within STS and medical sociology has promoted a resistance to defining what is ethical in favor of studying both how ethics are constituted in practice and, perhaps, what is at stake for those involved (Wainwright et al. 2006; Cribb et al. 2008; Pickersgill 2012; Hedgecoe 2004; Singleton 1996). Yet I propose that how we define actual ethical practices remains problematic. The distinction between normative frameworks and what scientists *themselves* saw as morally good or right (Wainwright et al. 2006) was conceptually useful, and, indeed, the interviewees engaged with prevailing moral or governance frameworks when describing their ethical dilemmas. Emphasizing the incompatibility or inadequacy of ethical guidelines, governance, and practices was often an interviewee’s way of articulating a desire for change or highlighting a lack of continuity. Moreover, work within ANT and the sociology of bioethics demonstrates that particular moral or governance tools are enacted in networks by particular configurations of patients, scientists, physicians, technologies, and artifacts (Timmermans and Berg 1997). However, as an analyst, I have found the distinction between the moral and the ethical *as constituted by conflict* to be more useful in enabling me to engage with ethics. Attending to controversy helps me demonstrate that ethics are not always embedded for the actors themselves. Rather, interviewees challenged and disagreed with current states of affairs by evoking connections to an idealized past or future or some other variety of elsewhere.

ANT approaches foreground ontological uncertainty through the empirical, compellingly arriving at the sorts of observations made by Law and Singleton (2005). The difference between that approach and the one I am suggesting here is perhaps a matter of degree in terms of engagement with the ontologically uncertain, or as I have suggested, foregrounding of the empirical. I have broadly agreed that any ethics that takes the empirical seriously should be more than the application of principles in particular settings. Still, the ethical, as I have tried to describe it, can be the province of the analyst at least as well as the actor. As I have attempted to demonstrate with my empirical examples, when engaging with the ethical the analyst and actor are equal in that they cannot rely entirely on what is immediately deployed, enacted, or actual. The reason for this is that in no situation in which ethical considerations arise is the “relation of cause and effect between actual things … a sufficient explanation” (Williams 2013, 56). There are at least two important implications of this for the argument I have advanced here. First, the actual––whether accessed through experiences or practices––is necessary but not sufficient for engagement with ethics. Second, insistence on a one-dimensional ontology––or a “deliberate actualism” (Harmon 2009)––creates an asymmetry between analyst and actor, excluding the latter from engaging in ethics.

Ethical moments we encounter in our empirical work confront both actor and analyst with “irresolvable problems that demand to be renewed rather than solved as classified and predictable” (Williams 2013, 183). This implies that, to paraphrase Fraser (2006), the analyst should be free to explore unrealized *ethical* potentialities. As we have seen, actors draw on ethical potentialities to argue that things need not be as they are but rather could and *should* be different. I have argued throughout for the removal of ontological barriers to analysts doing what is essentially the same thing: engaging with ethics.

Bringing together controversy and ethics, I have proposed a nonreductive and reflexive account of ethics in practice. My reading of the empirical data presented above supports an ontological structure comprising the virtual and the actual, which draws upon the strengths of ANT and sociology of bioethics in dealing with the empirical. These strengths include seeing controversy as providing useful insights in its own right and not simply as providing a focus for the problem-solving frameworks of moral philosophy. This is a case study of how to think “with Latour” (Lee and Stenner 1999) and other ANT proponents, and with the philosophy of Deleuze, about engagement with ethics and the empirical. I have run concepts together with data with the aim of creating a “conceptual hybridity” (Jensen 2014) that allows me to take both ethics and the empirical seriously. The interviewees raised questions of ethical interest, and the role of the analyst could be to propose ways in which to engage with the confrontations that the methods of STS open up to consideration. Ontologically, the actor network theorists leave the door open. My aim has been to venture through the door in relation to ethics in order to think of ethics neither as given in practices nor as fixed, eternal, and objective. In the ethics I am proposing, “there is neither certainty nor respite at any point” (Jun 2011, 104).

## Appendix

### Governing Genetic Databases—Topic Guide

#### Descriptive/factual information

##### About the interviewee

1. What is your professional background?
2. What is your function?
3. What kinds of samples/data are collected?
4. How did/do you obtain them?

- On what basis?

1. How big is the collection of samples/data?
2. For what purposes was the database designed?
3. Where does the funding for the project come from?
4. What do you call you collection of samples/data?

- Would you call your collection of samples/data a “genetic database”?

#### Insight into how GDBs are run

##### General

1. Professional Background
2. What are the origins of the database (who set it up and why)
3. Who besides the interviewee is involved in the management, design and use of the “GDB”?
4. How is the database used on a day-to-day basis?
5. What (if anything) has changed since the beginning of the project?
6. Which factors have most influence on practice?

##### Governance

1. Who is generally responsible for day-to-day management (whatever this means)?
2. What security measures are in place for the database?
3. Who can/could access the data?
4. How are decisions made about who accesses the data? (third parties)
5. Are there rules governing the design of genetic databases?
6. What is the consent process for inclusion in the study/database?
7. What are the main codes, laws, and guidelines governing practice?

- How has awareness of these affected practice?

1. Which official bodies are influential or relevant to practice?
2. At what stages have you thought about your responsibilities in law?

#### Opinion about future governance

1. What will be the needs of users of genetic databases in the future (say next ten years)?
2. What changes should be made to current guidance/governance systems?
3. What would an appropriate set of guidelines/legal instruments look like?
4. Would these alter with the type of database/collection/information?
5. Do current rules and guidelines reflect the requirements of users (or managers or others) of the database?
6. What will be the future uses of this sort of the collection/database you have built up?
7. Should data and biological samples be treated in a different way by the law? (What about extracted DNA or proteins?)

##### General

1. Description of a “GDB”

## Notes

1. Research ethics committees refer mostly to the Local Research Ethics Committees (LRECs), based in different National Health Service regions and tasked with approving research. The process of gaining ethics approval through LREC involved submitting the details of the proposed research, which would then be reviewed by the committee; the professional and lay memberships of these entities could vary. These processes were carried out under the auspices of the Central Office for Research Ethics Committees, now the Health Research Authority.

2. The interview was conducted with the leaders of the information technology team on the project at the same time due to their time constraints.

3. A meta-analysis involves looking across studies using statistical techniques to combine results.
